# Analytical Evaluation of the Ideal Strategy for High-Throughput Flow Injection Analysis by Tandem Mass Spectrometry in Routine Newborn Screening

**DOI:** 10.3390/metabo11080473

**Published:** 2021-07-22

**Authors:** Ilaria Cicalini, Silvia Valentinuzzi, Damiana Pieragostino, Ada Consalvo, Mirco Zucchelli, Simone Donzelli, Davide Ambrogi, Heather A. Brown, Lisa J. Calton, Liborio Stuppia, Vincenzo De Laurenzi, Piero Del Boccio, Claudia Rossi

**Affiliations:** 1 Center for Advanced Studies and Technology (CAST), “G. d’Annunzio” University of Chieti-Pescara, 66100 Chieti, Italy; ilaria.cicalini@unich.it (I.C.); silvia.valentinuzzi@unich.it (S.V.); d.pieragostino@unich.it (D.P.); ada.consalvo@libero.it (A.C.); m.zucchelli@unich.it (M.Z.); stuppia@unich.it (L.S.); delaurenzi@unich.it (V.D.L.); p.delboccio@unich.it (P.D.B.); 2 Department of Innovative Technologies in Medicine & Dentistry, “G. d’Annunzio” University of Chieti-Pescara, 66100 Chieti, Italy; 3 Department of Pharmacy, “G. d’Annunzio” University of Chieti-Pescara, 66100 Chieti, Italy; 4 Waters SPA, 20099 Milan, Italy; simone_donzelli@waters.com (S.D.); Davide_Ambrogi@waters.com (D.A.); 5 Waters Corporation, Scientific Operations, Wilmslow SK9 4AX, UK; Heather_A_Brown-Manchester@waters.com (H.A.B.); lisa_calton@waters.com (L.J.C.); 6 Department of Psychological, Health and Territorial Sciences, “G. d’Annunzio” University of Chieti-Pescara, 66100 Chieti, Italy

**Keywords:** metabolomics, newborn screening, metabolites, high-throughput omics technologies, mass spectrometry, inborn errors of metabolism, analytical evaluation

## Abstract

The introduction of tandem mass spectrometry (MS/MS) to clinical laboratories and the advent of expanded newborn screening (NBS) were crucial changes to public health programs worldwide. Speed, robustness, accuracy, selectivity, and specificity of analysis are all requirements of expanded NBS and are needed to minimize false positive results risks, to possibly eliminate false negatives, and to improve the positive predictive value of NBS. In this study, we firstly evaluated the analytical performances of the RenataDX Screening System, a fully integrated flow-injection MS/MS (FIA-MS/MS) IVD system for high-throughput dried blood spot (DBS) analysis in a routine NBS laboratory. Since a choice of several commercial NBS kits is available, we sought to compare NeoBase^TM^ 2 (PerkinElmer^®^) and MassChrom^®^ (Chromsystems) non-derivatized kits on the RenataDX platform by evaluating their analytical performances. Moreover, we verified the degree of correlation between data obtained by the two different NBS MS/MS kits by FIA-MS/MS of over 500 samples. Our data suggest that both methods correlate well with clinically insignificant differences that do not impact the NBS result. Finally, while NeoBase™ 2 offers an easier and faster sample preparation, MassChrom^®^ provides a cleaner sample extract which empirically should improve instrument reliability.

## 1. Introduction

Newborn screening (NBS) began during the 1960s when Robert Guthrie succeeded in developing a method for the detection of phenylketonuria (PKU) by a bacterial inhibition assay for the measurement of elevated concentrations of phenylalanine (Phe) in whole blood collected from infants by heel stick and dried onto special filter paper [1,2,3]. The introduction of tandem mass spectrometry (MS/MS) in clinical laboratories favored the expansion of NBS and the detection of more than 40 conditions using a single dried blood spot (DBS) sample [4,5,6,7,8]. Thanks to the ability to simultaneously quantify many analytes in a rapid analysis, MS/MS is well recognized as an innovative high-throughput technology with a multiplexing approach [9,10]. In fact, this crucial technology allowed to change the traditional screening approach of one-sample, one-test, one-marker, and one-disorder with one-sample, one-test, many markers, and many disorders [1]. It should be highlighted that the expansion of NBS, especially in the first decade of the 21st century, was one of the major changes to any public health program worldwide [11,12]. Indeed, a huge investment was made to potentiate NBS laboratories by including MS/MS technology [12]. NBS programs are quite different around the world. In fact, there is no global uniform screening panel, and the inborn errors of metabolism (IEMs) targeted by NBS vary between countries and sometimes also within regions [1]. It is well known that expanded NBS is not diagnostic but allows, through a biochemical profile, the identification of infants sufficiently at risk for a specific pathology. Expanded NBS is a complex multi- disciplinary system that plays a crucial role in preventive medicine—the ability to detect an IEM early, hopefully before the onset of significant and irreversible clinical effects, thus allowing immediate treatment in order to reduce morbidity and mortality [13,14,15]. In this view, expanded NBS perfectly fits with the P4 medicine (preventive, predictive, personalized, and participatory) model for infants with IEMs [16]. An expanded NBS test is required to be rapid yet robust and accurate and thus able to minimize the risk of false positive results, to possibly eliminate false negative results, and to improve the positive predictive value (PPV) of NBS results [15]. Moreover, an NBS test should be cost effective and easy to perform. Keeping in mind these requirements, it is clear that MS/MS offers speed of analysis, sensitivity and selectivity, and multiple metabolites to be monitored concurrently. In addition, the assay procedure is simplified by directly introducing the extracted samples into the source without chromatography [1], currently defined as flow injection analysis by MS/MS (FIA-MS/MS). All these requirements need to be met in expanded NBS, regardless of the MS/MS system used and the sample preparation procedure chosen.

In this study, we firstly evaluated the analytical performances of the RenataDX Screening System (Waters Corporation), a recently introduced FIA-MS/MS platform, for the clinical routine of NBS, also comparing the analytical parameters for the new FIA-MS/MS system with the laboratory’s existing and routinely used MS/MS system. Hence, considering that different in vitro diagnostic device (IVD) reagent kits for MS/MS NBS are commercially available, we also wondered what the pros and the cons of using one (IVD) kit rather than another would be with the goal of identifying the ideal analytical strategy in expanded NBS by FIA-MS/MS.

## 2. Results

### 2.1. Evaluation of the Analytical Performances between Acquity UPLC I-Class and Renatadx Screening Systems: Study 1

The repeatability estimated for both the analytical systems by analyzing 36 QC levels (18 low QCs and 18 high QCs) from the NeoBase™ 2 Non-derivatized MSMS kit is listed in Table 1 as %CV. Repeatability values were found to be within the 10% of accepted variable limits for each QC level analyzed on both analytical platforms. By comparing the repeatability between the two different technologies, it was shown that RenataDX Screening system was associated with lower %CV values for 11/20 analytes related to the low QC level, whereas the lower %CV values related to the high QC level were likewise pointed out for 11/20 analytes estimated by ACQUITY UPLC I-Class system, thus proving a comparable repeatability.

The intra-day precision evaluated for both the platforms by analyzing six replicate analyses of four QC levels (A, B, C, D) provided by Centers for Disease Control and Prevention (CDC) is reported in Supplementary Table S1 as %CV. The inter-day precision for the two analytical systems estimated as %CV by analyzing each QC level provided by CDC over a 5 day period is listed in Supplementary Table S2. The intra-day %CV values were found to be within the 10% for all the QC levels on both the systems, except for C14 (11.1%), C6 (15.7%), and C5DC\C6OH (22%) from level A analyzed by ACQUITY UPLC I-Class platform. In the same context, the inter-day %CV values were found to be within 10% for all the QC levels on both the analytical platforms, except for Cit (12.9%) and C5DC\C6OH (19.5%) from level A and C6 (11.7%) from level C analyzed by ACQUITY UPLC I-Class platform. The comparison between the %CV values obtained with the two different technologies shows that a greater number of analytes (12/20) was associated with lower %CV values for QC level A by using ACQUITY UPLC-I Class system, although some repeatability values exceeded the accepted limits contrary to the perfect agreement displayed by RenataDX Screening System; QC level B was associated with an equivalent number of lower %CV values produced by the comparison between ACQUITY UPLC I-Class and RenataDX. On the other hand, QC levels C and D were associated with lower %CV values for 12/20 and 11/20 analytes, respectively, by using RenataDX Screening System. In the same way, the comparison of the inter-day repeatability values obtained with the two different systems reveals that RenataDX Screening platform produced the lower %CV values for 12/20, 13/20, 16/20, and 11/20 analytes from levels A, B, C and D, respectively.

Carryover from nine plates analyzed by both the analytical systems was also assessed. Supplementary Table S3 shows that carryover values were all within 0.25% and 0.48% by ACQUITY UPLC I-Class and RenataDX Screening technologies, respectively. The comparison between the values derived from the two platforms reveals that RenataDX Screening System exhibited lower carryover effects, except for seven analytes that had no signs of carryover when using the ACQUITY UPLC I-Class system.

### 2.2. Methods Comparison between ACQUITY UPLC I-Class and RenataDX Systems

**Figure 1:**
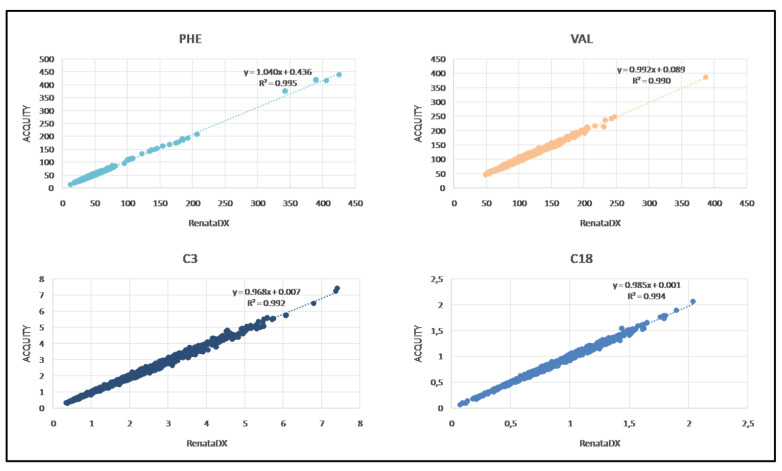
Correlation curves of Phe and Val as aminoacids and C3 and C18 as acylcarnitines, showing the equation of the linear regression analysis and their respective R^2^ values.

To assess the linear agreement between the two analytical platforms, a correlation coefficient was calculated for each analyte by plotting its concentration obtained by analyzing 1092 neonatal DBS samples on both ACQUITY UPLC I-Class and RenataDX Screening systems. As summarized in Supplementary Table S4, R^2^ values were found greater than or equal to 0.95 for all the analytes, except for C5DC\C6OH (0.78), C6 (0.86), Cit (0.89), and C8 (0.90). Figure 1 shows an example of the linear association between the systems for Phe, Val, C3, and C18 with the respective linear regression analysis and correlation coefficient.

### 2.3. Evaluation of Two Commercial MS/MS Kits Used Rountinely in Newborn Screening on the RenataDX Screening System: Study 2

#### 2.3.1. Linearity of the MassChrom^®^ LC-MS/MS Kit

Linearity for MassChrom^®^ LC-MS/MS kit was assessed by analyzing three replicates of 11 levels provided by the kit itself. The regression analysis confirmed the linear trend of the method as ruled by Food and Drug Administration (FDA) guidelines, showing that the coefficient of determination R^2^ was greater than or equal to 0.995 for all the analytes, with the exception for Arg (0.991) and C5DC\C6OH (0.994), as listed in Table 2. Supplementary Figure S1 shows the linear regression curves for some analytes of interest.

#### 2.3.2. Evaluation of the Analytical Performances between NeoBase™ 2 and MassChrom^®^ Non-Derivatized Kits

The intra-day precision evaluated for both the methods by analyzing three replicate analyses of two QC levels (high and low) provided by each manufacturer is summarized in Table 3 as %CV. The inter-day precision for the two NBS kits estimated as %CV by analyzing each QC level provided by the manufacturers over a 9 day period is listed in Table 4. The intra-day %CV values were found to be within the 10% for all the QC levels by both NBS methods, except for C5DC\C6OH (11.1%) from high QCs processed by NeoBase™ 2, C4 (10.4%), C5DC\C6OH (15.1%), C8 (10.6%), C14 (11%), C16 (12.7%), and C18 (14.1%) from high QCs analyzed by MassChrom^®^. The inter-day %CV values were found to be within the 10% for all the QC levels processed by both analytical methods, except for C5DC\C6OH (12.2%) from low QCs analyzed by NeoBase™ 2 kit. The comparison between the %CV values obtained with the two different NBS kits highlights that an equal intra-day repeatability for low QC levels was achieved by using both the methods; conversely, a greater number of lower %CV values was achieved for 17/20 analytes by processing high QC levels with NeoBase™ 2kit. In the same way, the comparison between the inter-day repeatability values reveals that MassChrom^®^ kit was associated with the lower %CV values for both the low (15/20 analytes) and the high (11/20 analytes) QC levels.

**Figure 2:**
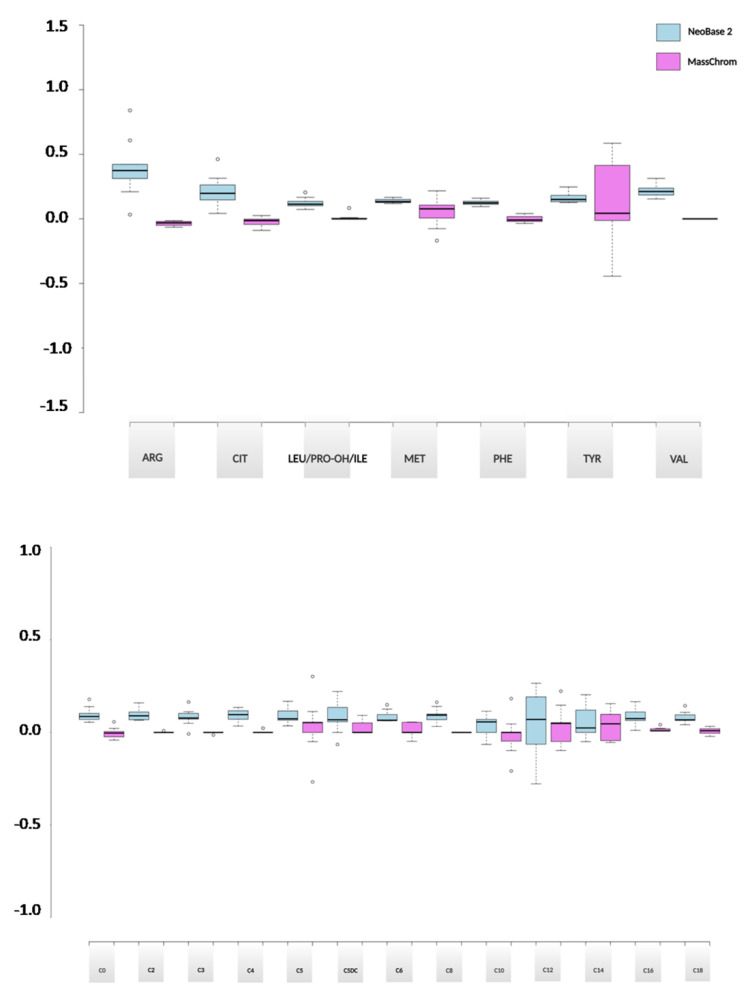
Carryover and SD values visualized by box plots for amino acids (top panel) and acylcarnitines (bottom panel). For each analyte, blue and violet colors indicate NeoBase™ 2 and MassChrom^®^ non-derivatized kits, respectively. (SD: standard deviation).

Carryover effect was established taking into account the blanks and the QC levels over a 9 day period through the comparison of the values given by the two NBS kits. Carryover values were found to be within 0.4% for all the analytes. Overall, MassChrom^®^ kit was associated with a lower carryover effect than NeoBase™ 2, as shown in Supplementary Table S5 and summarized in Figure 2 by box plots for both aminoacids and acylcarnitines.

#### 2.3.3. Methods Comparison between NeoBase^™^ 2 and MassChrom^®^ Non-Derivatized Kits

**Figure 3:**
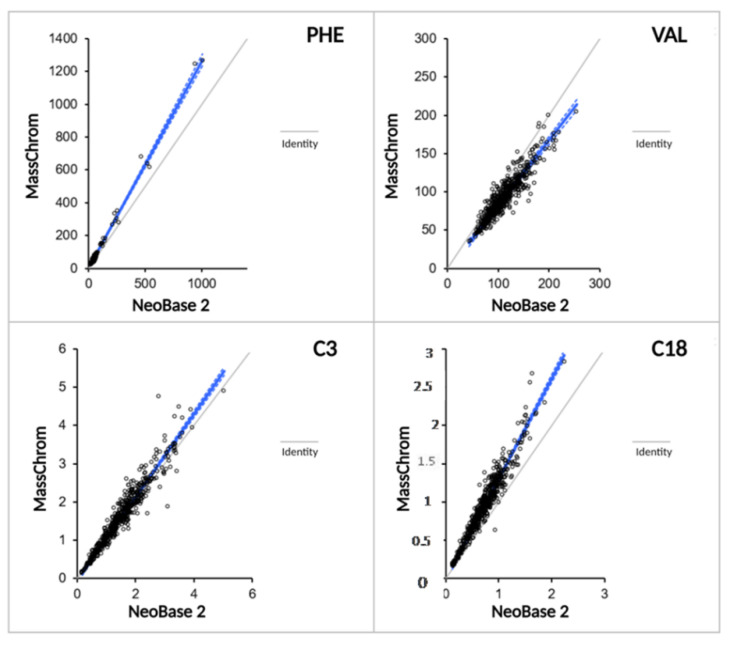
Scatter plots by Passing–Bablok for Phe, Val, C3, and C18 after method comparison between NeoBase™ 2 and MassChrom^®^ non-derivatized kits.

**Figure 4:**
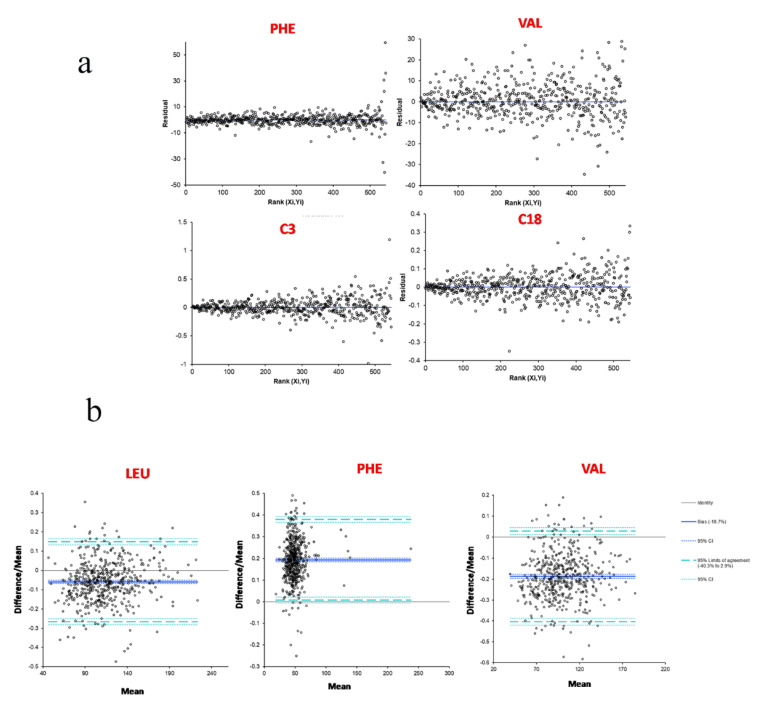
(**a**) Residual plots for Phe, Val, C3, and C18 after method comparison between NeoBase™ 2 and MassChrom^®^ non-derivatized kits; (**b**) difference plots for Leu, Phe, and Val after the removal of DBS samples from diagnosed IEMs subjects.

Methods comparison results using Passing–Bablok fit (Analyse-it) after 543 neonatal DBS samples were processed with both NBS kits are summarized in Table 5 as slope and intercept values of the regression curves with their 95% CI. Figure 3 shows the resulting scatter plots for some analytes of interest. The bias plots provided the random differences that can be expected between the two methods for each analyte: the residual standard deviations (RSDs) of aminoacids were found to be lower or equal to 20, whereas those of acylcarnitines were extremely low (<8). Figure 4a shows the bias plots for some analytes of interest. Leu, Phe, and Val were associated with RSD values greater than the acceptable or the reasonable limit for clinical purposes when including DBS samples from diagnosed IEMs patients; finally, the removal of those samples restored the acceptability of the residual differences between the methods (Figure 4b).

## 3. Discussion

In our study, taking in mind speed, robustness, accuracy, selectivity, and specificity of analysis as required for expanded NBS, we initially evaluated the analytical performances of the RenataDX Screening System for high-throughput DBS analysis in our NBS laboratory. Once it was verified that the RenataDX Screening System met the aforementioned needs of NBS, we further evaluated the use of NeoBase™ 2 and MassChrom^®^ non-derivatized kits on the RenataDX platform in routine MS/MS NBS. The first study was designed to compare different analytical platforms specifically designed (or not) for supporting high throughput newborn screening on DBS samples via FIA-MS/MS. On one hand, the coupling of the ACQUITY UPLC I-Class system with a Xevo TQD mass spectrometer is already largely employed for NBS purposes, mainly due to the fact that the resulting platform can be adapted either to flow injection analysis or to second-tier tests via chromatographic separation. On the other hand, the most recent technology developed by Waters Corp. in the field of NBS, named RenataDX Screening System, meets the demands of analytical simpleness, automaticity, and robustness for large scale screening by coupling its compact architecture to the 3777C IVD Sample Manager, the ACQUITY UPLC I-Class IVD Binary Solvent Manager, and the Xevo TQD IVD mass spectrometer.

To confirm the diagnostic power of the RenataDX Screening System on DBS samples for expanded NBS, the NeoBase™ 2 Non-derivatized MSMS kit was used to measure amino acids and acylcarnitines by MS/MS analysis on both platforms, taking into consideration that its analytical performances were already verified on the ACQUITY UPLC I-Class system coupled to the Xevo TQD. Repeatability established by analyzing QC levels provided both by CDC and the kit itself was comparable between the two systems and was found to comply with the accepted variable limits. Moreover, carryover effects were generally low on both analytical platforms, although the best results were obtained by RenataDX Screening System. These data combined with the ones derived from the correlation analysis corroborate the reliability of the RenataDX Screening System as one possible technology with high flexibility, robustness, and ease of use for high-throughput NBS analysis on DBS samples. In this way, the NBS laboratory staff can implement their clinical programs with the same confidence that is currently already guaranteed by the ACQUITY UPLC I-Class platform. This study could support them in their choice of analytical system to better meet their needs. Taking the performances for granted, the RenataDX Screening System supplies large scale NBS, whereas the ACQUITY UPLC I-Class system can also accommodate second-tier tests beyond the first screening when coupled to LC technologies.

It is indeed important to note that expanded NBS by tandem mass spectrometry is normally performed directly by a flow injection analysis and, since it does not require prior chromatographic separation, potential interferers as well as the matrix effect could occur [17]. In order to overcome these limits while maintaining the typical characteristics required for screening tests, second-tier testing is performed in newborns with abnormal screening results. In this context, quantitative methods for acylcarnitines and amino acids using high resolution chromatography and tandem mass spectrometry are suitable as second-tier tests for newborn screening of specific disorders associated with abnormal levels of acylcarnitines and amino acids, potentially reducing false positive cases and shortening the time to diagnosis [17,18]. The second study was designed to evaluate different commercial NBS kits for FIA-MS/MS of DBS samples on the RenataDX Screening System. The relevance of this study is derived from the fact that NBS worldwide does not have the same programs [19], thus the possibility to consciously choose among the different available commercial kits guarantees a compromise in terms of costs, sample preparation, and metabolites coverage that relies on the specific panel of diagnosed IEMs. Our experience let us compare NeoBase™ 2 and MassChrom^®^ non-derivatized kits by evaluating their repeatability and carryover effects. NeoBase™ 2 tests for more than 50 analytes, including the markers of adenosine deaminase severe combined immunodeficiency (ADA-SCID) and X-linked adrenoleukodystrophy (X-ALD), following a fast extraction procedure that allows also for the yield of diketones (e.g., succinylacetone). On the other hand, MassChrom^®^ ensures extremely low interferences due to the use of filter plates, guaranteeing the possibility to extract diketones as well with a simple additional step. For our purposes, only the shared analytes were actually considered, although succinylacetone was removed from the list since it undergoes different extraction procedures and MRM acquisitions. Since linearity and precision were already determined for NeoBase™ 2 kit, as reported in the manufacturer’s certification, MassChrom^®^ kit was firstly validated in terms of linearity, thus adding values to the analytical reproducibility stated among the submitted information. Then, an evaluation of the analytical performances between the two kits was done to highlight their similarities and possible pros and cons. In this context, it was clear that both analytical methods met the demands of repeatability and carryover effects with values that were always found to be within the accepted limits. More interestingly, carryover was overall lower when using MassChrom^®^ kit, as expected by the use of the filtering plates that minimized the cross-contamination, also providing a cleaner sample extract, which empirically should improve instrument reliability. Importantly, it is strongly recommended that operators must be aware of the necessity to routinely update specific response factors (RRF) that become relevant to correct the response as a function of the sensitivity of the mass spectrometer. Our comparison was made considering RRF values of one for each analyte measured by both kits, thus normalizing the responses on the same conditions for quantification. It is also important to note that some analytes such as C5DC\C6OH showed worse performance, although still fully acceptable, in terms of linearity and intra-day precision. Specifically, this analyte was the result of the summation of the two structural isomers (C5DC and C6OH) and, probably for this reason, the measurement was less accurate than the other analytes.

In fact, DBS acylcarnitine profiling is a useful method for high-throughput newborn screening of metabolic disorders, but differentiation of isobaric and isomeric compounds is not achievable, especially for laboratories that routinely use non-derivatized methods for acylcarnitines quantification [17].

In order to improve and overcome the problems due to the simultaneous quantification of these isobaric species (C3DC\C4OH, C4DC\C5OH, and C5DC\C6OH), the use of second tier test is strongly recommended. These methods can rely on sample derivatization processes or be based on the use of high-resolution mass spectrometers with a prior separation by liquid chromatography [17,20,21]. Finally, the method comparison performed on DBS samples supported the high degree of correlation between NeoBase™ 2 and MassChrom^®^ kits, highlighting residual differences that were always found within the reference limits for clinical purposes. Some analytes (Leu, Phe, Val) exceeded the RSD values for which the interchangeability of the methods could be reasonably considered, but this occurred taking into account DBS samples from diagnosed IEMs subjects. Actually, results could be equally associated with clinical effectiveness, since the presence of extremely high concentrations of analyte—far from the linearity range, the cut-offs, and the QC levels normally used for measurements—necessarily provokes a strong deviation from the mean values. Assuming that MS/MS NBS is not a diagnostic test but rather a clinical tool that, through a biochemical profile, is able to identify newborns potentially at risk for a given IEM, NBS provides a suspicion of IEMs when values exceed the reference limits. Thus, exceeding the cut-off is a sufficient condition to establish a diagnostic suspicion, even if the quantification is not directed in the linear range. Leu, Phe, and Val were reprocessed after excluding those DBS samples from affected patients, thus restoring the acceptability of the residual differences between the methods. These data further validate the high suitability of both NeoBase™ 2 and MassChrom^®^ non-derivatized kits in MS/MS NBS, also confirming their comparable values in terms of reproducibility, carryover, and degree of correlation. Finally, the last words are up to the NBS laboratory staff who could make a list of pros and cons for two available kits: NeoBase™ 2 perfectly fits for rapid applications in high-throughput screening programs, while MassChrom^®^ elegantly guarantees low interferences and is suggested for fully automated sample preparation systems. Once again, their choice relies on their laboratory demands in terms of speed, simplicity, metabolite coverage, and costs. In conclusion, it is well known that NBS tests performed by immunoassay techniques as well as by digital microfluidic systems still suffer from high false positive rates. Hence, all the described aspects acquire even more value since MS/MS, also thanks to its multiplexing approach, speed of analysis, selectivity, and sensitivity, should remain the method of choice in expanded NBS for a long time [1].

## 4. Materials and Methods

### 4.1. FIA-MS/MS for the Comparison between Two Analytical Platforms

**Figure 5:**
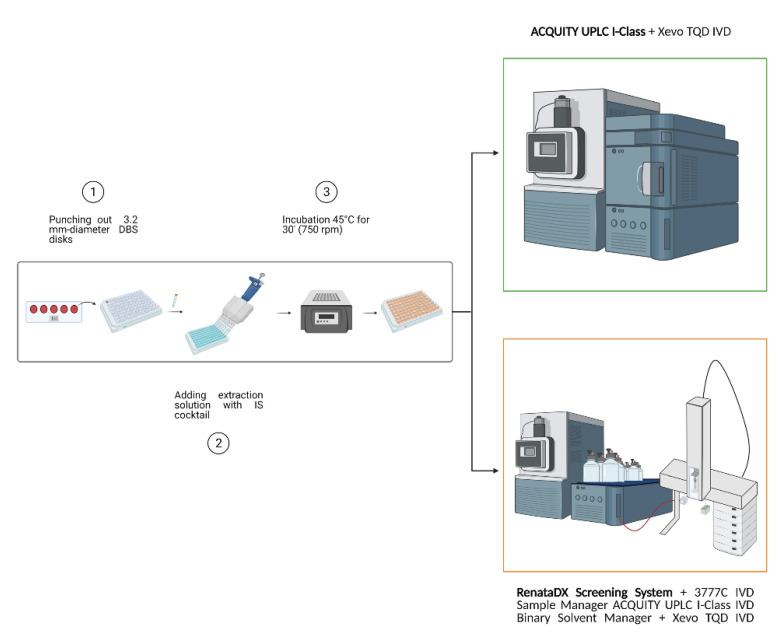
Workflow of sample preparation using NeoBase™ 2 Non-derivatized MSMS kit and parallel analysis by ACQUITY and RenataDX systems. Image created with BioRender.com.

The flow injection analysis tandem mass spectrometry (FIA-MS/MS) of a metabolite profile in dried blood spot (DBS) samples was performed using two different systems, both with a Xevo™ TQD IVD Mass Spectrometer (Waters Corporation, Milford, MA, USA). The first system consisted of an ACQUITY UPLC I-Class coupled to a Xevo TQD IVD tandem quadrupole mass spectrometer (Waters Corporation, Milford, MA, USA) that is widely used not only for FIA-MS/MS approaches but also for column chromatography, whereas the second system consisted of a RenataDX Screening System including 3777C IVD Sample Manager, ACQUITY™ UPLC™ I-Class IVD Binary Solvent Manager, and Xevo™ TQD IVD Mass Spectrometer (Waters Corporation, Milford, MA, USA). In Figure S2, examples of a typical flow injection chromatogram are shown for both instrumental setups used. In both cases, the MS system operated in positive electrospray ionization mode using multiple reaction monitoring (MRM) as a targeted approach for signal acquisition. MS parameters are listed in Supplementary Table S6 considering MS/MS transitions, cone potentials, and collision energies for each metabolite and its relative internal standard. FIA-MS/MS analysis was performed on 1092 neonatal DBS samples, quality controls (QCs) provided by NeoBase™ 2 Non-derivatized MSMS kit (PerkinElmer Life and Analytical Sciences, Turku, Finland), and QCs from CDC (Centers for Disease Control and Prevention). DBS samples were punched out into 3.2 mm disks and extracted following the manufacturer’s instructions of the NeoBase™ 2 kit. Figure 5 shows the operative workflow from the DBS sample preparation to the analysis of the two analytical platforms. In total, 10 µL of extracted samples were injected into the ion source with a run time of 1.1 min injection-to-injection. The mobile phase was kindly provided by the diagnostic kit in use. Data were processed by MassLynx™ (IVD) Software V4.2 with NeoLynx™ Application Manager or with IonLynx Application Manager (Waters Corp.).

### 4.2. Evaluation of the Analytical Performances between ACQUITY UPLC I-Class and RenataDX Systems

Repeatability in terms of inter-day and intra-day precision was evaluated for both systems in order to quantify the degree of agreement between independent measurements under selected conditions of use. Repeatability test was also performed through the analysis of QC materials from the NeoBase™ 2 Non-derivatized MSMS kit, quantifying the %CV of 18 replicate analyses at two different concentration levels per analyte. To investigate the agreement between the two analytical systems in comparison, the correlation coefficient was estimated as an indicator of the quality of the data through the analysis of 1092 DBS samples by ACQUITY UPLC I-Class-Xevo TQD and RenataDX Screening systems. In this context, no other recommended analytical tools for method comparison were adopted, since great correlation coefficients generally come with data that are not biased by the error in the variable. Moreover, to assess the degree of contamination between consecutive measurements, carryover was calculated for both analytical platforms (Figure S3). Details of the statistical methods used in the evaluation of analytical performances between ACQUITY UPLC I-Class and RenataDX systems are fully described in Section S1 of Supplementary Materials.

### 4.3. Linearity of the MassChrom^®^ LC-MS/MS Kit

The linearity of the MassChrom^®^ amino acids and acylcarnitines from dried blood/non-derivatized LC-MS/MS kit (Chromsystems Instruments & Chemicals GmbH, Gräfelfing/Munich, Germany) was assessed by 3 replicate analyses of 11 different concentration levels per analyte within 3 days. In this study, the technology consisted of a RenataDX Screening System comprising 3777C IVD Sample Manager, ACQUITY™ UPLC™ I-Class IVD Binary Solvent Manager, and Xevo™ TQD IVD Mass Spectrometer (Waters Corporation, Milford, MA, USA). Linearity was evaluated in terms of correlation coefficient R^2^ that was calculated as the square of the Pearson correlation coefficient derived from the equation linking the expected concentrations provided by the manufacturer’s datasheet (as X variable) to the observed ones (as Y variable).

### 4.4. Extraction of DBS Samples by NeoBase™2 Kit

DBS samples were punched out into 3.2 mm diameter disks to get the extraction of amino acids and acylcarnitines. As previously described [22,23,24], each DBS spot was incubated with internal standard (IS) solution for 30′, 700 rpm, +45 °C. Finally, 10 µL of supernatant was injected into the ion source, and the run time was 1.1′ injection-to-injection. The flow with the mobile phase provided by the kit was set at 0.150 mL/min, and the flow gradient was set as follows: 0.15 mL/min from 0 to 0.17′; 0.01 mL/min from 0.17′ to 0.98′; 0.7 mL/min from 0.98′ to 1.18′; 0.15 mL/min from 1.18′ to the end. Details of MS operation mode were reported above, and MS parameters are listed in Supplementary Table S6. Processing of the data was carried out by MassLynx™ (IVD) Software V4.2 with IonLynx™ Application Manager (Waters Corp., Wilmslow, UK). Actually, the NeoBase™ 2 Non-derivatized MSMS kit is designed for the quantification of many other analytes and, more interestingly, of succinylacetone (SA), whose yield from DBS samples is guaranteed by incubating the supernatant after extraction for 60′ at RT before injection.

### 4.5. Extraction of DBS Samples by MassChrom^®^ Kit

DBS samples were punched out into 3.2 mm diameter disks for the extraction of amino acids and acylcarnitines. Each DBS disk was located in a well of a filtering multiplate put on top of a collection multiplate and incubated with IS solution for 20′ at 600 rpm. The supernatant in the filtering multiplate was centrifuged for 7′ and 3000× *g* and was collected in the multiplate below. Finally, 10 µL from the collection multiplate were injected into the ion source. The run time was 1.3′ injection-to-injection. The flow with the mobile phase provided by the kit was set at 0.250 mL/min, and the flow gradient was optimized as follows: 0.25 mL/min from 0 to 0.31′; 0.04 mL/min from 0.31′ to 1.15′; 0.8 mL/min from 1.15′ to 1.25′; 0.25 mL/min from 1.25′ to the end. The MS system operated in positive electrospray ionization mode using multiple reaction monitoring (MRM) as a targeted approach for signal acquisition. MS parameters are listed in Supplementary Table S7 considering MS/MS transitions, cone potentials, and collision energies for each metabolite and its relative internal standard. Data were processed by MassLynx™ (IVD) Software V4.2 with IonLynx™ Application Manager (Waters Corp.). SA was not included in the analysis, even though it can be extracted by incubating the residual DBS disk in the filtering multiplate with a proper solution for 30′, 600 rpm, and +45 °C and by recombining the two extracts after another centrifugation step.

### 4.6. Evaluation of the Analytical Performances between NeoBase™ 2 and MassChrom^®^ Non-Derivatized Kits

**Figure 6:**
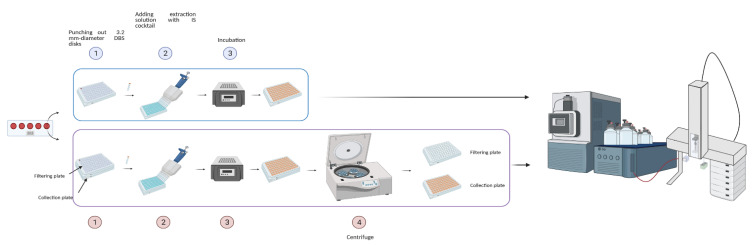
Workflow of sample preparation using NeoBase™ 2 (on the top panel remarked in light blue) and MassChrom^®^ (on the bottom panel remarked in light violet) non-derivatized kits by parallel analysis on the RenataDX Screening System. Image created with BioRender.com.

The intra-day precision for both NBS kits by FIA-MS/MS with RenataDX Screening System was established by analyzing 3 replicate analyses of two QCs levels (high and low) provided by each manufacturer. On the other hand, the inter-day precision was estimated by analyzing each QC level provided by the manufacturers over a 9 day period. Finally, the precisions established respectively for the NeoBase™ 2 kit and for the MassChrom^®^ kit were directly compared by highlighting the lower %CV per analyte. In order to define the possible degree of contamination between consecutive measurements, carryover was estimated for both methods. The carryover was measured by analyzing nine 96 well plates, and the carryover values obtained by MassChrom^®^ and NeoBase™ 2 kits were compared to establish the best solution to minimize cross-contamination during FIA-MS/MS analysis (Figure S3). Figure 6 shows the operative workflow of the study 2.

### 4.7. Methods Comparison

Data derived by FIA-MS/MS analysis of 543 neonatal DBS samples extracted using both NeoBase™ 2 and MassChrom^®^ kits were processed by Analyse-it. As mentioned before, SA was not considered for method comparison because it undergoes different extraction procedures among the NBS kits, and it is also monitored by different MRM transitions. No verification of data distribution was done due to the large size of the dataset that could directly justify the approximation to normality, as explained by the central limit theorem. To verify the degree of correlation between data respectively obtained by NeoBase™ 2 and MassChrom^®^ kits, a correlation analysis was provided by Passing–Bablok regression (Analyse-it) that was performed as a non-parametric test to estimate the linear regression line of X and Y biased variables. In the Passing–Bablok equation, the intercept gives a measure of the systematic bias between the two methods, while the slope quantifies the amount of proportional bias between them. The confidence levels were set to 95%. Bias plots were supplied to show if 95% of the residuals lied in the interval ±1.96 times the residual standard deviation (RSD), thus defining the random differences between the kits. In this way, an assumption on the interchangeability of the methods based on mutual acceptable differences could be expressed.

## Figures and Tables

**Table 1:** Comparison of repeatability values from NeoBase™ 2 Non-derivatized MSMS kit QC levels between ACQUITY UPLC I-Class and RenataDX Screening systems. For each analyte, the lower %CV value was highlighted in bold for both low (LC) and high (HC) QC levels comparison.

Repeatability	ACQUITY		RenataDX	
	LC CV%	HC CV%	LC CV%	HC CV%
ARG	**6.15**	**5.08**	6.17	5.66
CIT	**8.81**	8.43	9.73	**7.48**
LEU\ILE\PRO-OH	6.47	6.90	**6.02**	**6.85**
MET	**7.72**	**9.26**	9.40	9.68
PHE	7.20	**7.19**	**6.87**	7.28
TYR	7.43	**6.29**	**7.28**	6.41
VAL	5.09	5.45	**4.86**	**4.42**
C0	8.89	8.18	**5.03**	**5.29**
C10	**6.74**	7.71	6.93	**6.37**
C2	**3.72**	4.93	4.20	**4.88**
C3	**4.72**	4.52	5.59	**3.32**
C4	6.04	**6.32**	**5.93**	6.60
C5	5.47	5.28	**5.03**	**5.19**
C5DC\C6OH	9.86	**6.77**	**8.36**	7.99
C6	**6.20**	**4.58**	6.66	5.08
C8	5.71	**5.59**	**5.30**	5.90
C12	6.53	**6.20**	**5.42**	6.48
C14	**5.47**	**6.12**	5.77	6.41
C16	4.79	6.29	**4.76**	**5.56**
C18	**5.92**	**7.65**	7.96	9.97

**Table 2:** R^2^ and SD values from linearity validation by the analysis of 11 levels provided by MassChrom^®^ kit on the RenataDX Screening System (SD: standard deviation).

	R^2^	SD
ARG	0.991	0.007
CIT	0.996	0.001
LEU\ILE\PRO-OH	0.996	0.002
MET	0.997	0.002
PHE	0.997	0.001
TYR	0.998	0.001
VAL	0.996	0.001
C0	0.995	0.003
C10	0.997	0.002
C2	0.997	0.001
C3	0.997	0.002
C4	0.998	0.001
C5	0.997	0.002
C5DC\C6OH	0.993	0.001
C6	0.997	0.001
C8	0.997	0.002
C12	0.997	0.002
C14	0.998	0.001
C16	0.998	0.001
C18	0.998	0.0007

**Table 3:** Comparison of intra-day precision values from QC levels provided by manufacturers between NeoBase™ 2 and MassChrom^®^ non-derivatized kits on the RenataDX Screening System. For each analyte, the lower %CV value was highlighted in bold when comparing low and high QCs, respectively.

*n* = 3	NeoBase™ 2		MassChrom^®^	
	LC CV%	HC CV%	LC CV%	HC CV%
ARG	**1.93**	4.76	8.21	**0.46**
CIT	**2.50**	7.37	2.74	**6.72**
LEU\ILE\PRO-OH	**0.85**	**5.28**	1.49	8.65
MET	3.29	**4.47**	**2.24**	9.35
PHE	1.84	**4.19**	**1.29**	8.49
TYR	3.57	**5.71**	**1.57**	7.59
VAL	**0.70**	**6.18**	1.94	8.69
C0	2.23	**5.36**	**1.89**	8.84
C10	5.15	8.07	**0.81**	**5.89**
C2	1.59	**5.49**	**0.98**	8.41
C3	2.60	**4.84**	**1.50**	10.00
C4	4.53	**6.09**	**2.92**	10.40
C5	**2.35**	**3.30**	3.39	8.10
C5DC\C6OH	**3.90**	**11.14**	9.02	15.07
C6	6.00	**4.82**	**3.73**	6.57
C8	**1.39**	**3.56**	3.88	10.56
C12	5.07	**8.18**	**2.70**	8.98
C14	**1.02**	**5.05**	1.31	11.01
C16	**1.63**	**5.74**	1.88	12.65
C18	**0.30**	**7.15**	1.76	14.08

**Table 4:** Comparison of inter-day precision values from QC levels provided by manufacturers between NeoBase™ 2 and MassChrom^®^ non-derivatized kits on the RenataDX Screening System. For each analyte, the lower %CV value was highlighted in bold when comparing low and high QCs, respectively.

*n* = 9	NeoBase™ 2		MassChrom^®^	
	LC CV%	HC CV%	LC CV%	HC CV%
ARG	**4.75**	3.57	7.04	6.49
CIT	9.29	8.22	**7.09**	**6.14**
LEU\ILE\PRO-OH	5.31	**4.29**	**4.24**	4.98
MET	**4.43**	**4.75**	5.61	5.47
PHE	4.09	**3.74**	**4.02**	5.22
TYR	5.33	6.28	**3.05**	**5.76**
VAL	6.93	**4.77**	**4.36**	5.36
C0	5.84	5.60	**5.20**	**5.03**
C10	8.92	7.25	**5.41**	**5.43**
C2	7.11	6.39	**4.17**	**5.48**
C3	9.61	6.38	**5.10**	**5.33**
C4	6.45	6.27	**6.01**	**5.99**
C5	7.62	5.25	**4.79**	**5.13**
C5DC\C6OH	12.15	7.44	**6.59**	**5.97**
C6	**4.21**	5.34	4.37	**4.73**
C8	7.43	**4.89**	**5.10**	4.97
C12	8.40	**5.69**	**3.47**	7.02
C14	**4.92**	**2.54**	5.45	5.99
C16	9.78	8.19	**4.94s**	**7.46**
C18	**6.56**	**4.22**	6.65	8.63

**Table 5:** Slope and intercept values of the regression curves for each analyte after method comparison between NeoBase™ 2 and MassChrom^®^ non-derivatized kits by Passing–Bablok.

Passing-Bablok Fit	Slope	Intercept
ARG	0.95	−0.92
CIT	1.08	−0.92
LEU\ILE\PRO-OH	1	−6.05
MET	0.94	0.17
PHE	1.26	−2.04
TYR	1.10	0.62
VAL	0.86	−3.37
C0	0.98	0.16
C2	1.03	−0.48
C3	1.08	−0.04
C4	1.13	−0.01
C5	0.89	0
C5DC\C6OH	1.84	−0.01
C6	1.03	−0.01
C8	1.09	−0.02
C10	1.52	−0.01
C12	1.11	0
C14	1.02	0
C16	1.12	0
C18	1.31	−0.01

## Data Availability

Data is contained within the article and supplementary materials.

## Supplementary Materials

The following are available online at https://www.mdpi.com/article/10.3390/metabo11080473/s1, Figure S1: Linearity curves from the analysis of the third replicate of Phe and Val as aminoacids, C3 and C18 as acylcarnitines, showing the equation of the linear regression curves and their respective R^2^ values, Figure S2: Examples of a typical flow injection chromatogram obtained by analyzing a low quality control (LC) provided by NeoBase™ 2 Non-derivatized MSMS kit on the RenataDX Screening System (A) and the ACQUITY UPLC I-Class System (B), Figure S3: Zoom of a canonical 96-well plate to show how it is prepared for NBS, starting with a first blank that is immediately followed by low QC, high QC and a second blank, Table S1: Comparison of intra-day precision values from CDC QC levels between ACQUITY UPLC I-Class and RenataDX Screening systems, Table S2: Comparison of inter-day precision values from CDC QC levels between ACQUITY UPLC I-Class and RenataDX Screening systems, Table S3: Comparison of carryover effects between ACQUITY UPLC I-Class and RenataDX Screening systems, Table S4: R2 values from correlation analysis of DBS samples between ACQUITY UPLC I-Class and RenataDX Screening systems, Table S5: Comparison of carryover effects by NeoBase™ 2 and MassChrom^®^ non-derivatized kits on the RenataDX Screening System, Table S6: MS parameters used for FIA-MS/MS analysis by NeoBase™ 2 Non-derivatized MSMS kit, Table S7: MS parameters used for FIA-MS/MS analysis by MassChrom^®^ Amino Acids and Acylcarnitines from Dried Blood/Non derivatized LC-MS/MS kit.
