# Supplementary material for: Analytical Evaluation of the Ideal Strategy for High-Throughput Flow Injection Analysis by Tandem Mass Spectrometry in Routine Newborn Screening
Source: Metabolites. 2021 Jul 22;11(8):473. doi: 10.3390/metabo11080473 (PMC8399422; doi:10.3390/metabo11080473)
Supplement: Supplementary file 1 [file metabolites-11-00473-s001.zip › metabolites-1300721-supplementary.pdf]

Article

Supplementary of: Analytical evaluation of the ideal strategy for High-throughput flow injection analysis by tandem mass spectrometry in routine newborn screening

Ilaria Cicalini[1,2§], Silvia Valentinuzzi[1,3§], Damiana Pieragostino[1,2], Ada Consalvo[1,2], Mirco Zucchelli[1,2], Simone Donzelli[4], Davide Ambrogi[4], Heather A. Brown[5], Lisa J. Calton[5], Liborio Stuppia[1,6], Vincenzo De Laurenzi[1,2], Piero Del Boccio[1,3], and Claudia Rossi[1,6*]

[1] Center for Advanced Studies and Technology (CAST), "G. d'Annunzio" University of Chieti-Pescara, 66100 Chieti, Italy; ilaria.cicalini@unich.it; silvia.valentinuzzi@unich.it; d.pieragostino@unich.it; ada.consalvo@libero.it; m.zucchelli@unich.it; stuppia@unich.it; delaurenzi@unich.it; p.delboccio@unich.it; claudia.rossi@unich.it.

[2] Department of Innovative Technologies in Medicine & Dentistry,"G. d'Annunzio" University ofChieti-Pescara, 66100 Chieti, Italy.

[3] Department of Pharmacy, "G. d'Annunzio" University of Chieti-Pescara, 66100 Chieti, Italy;

[4]Waters SPA, 20099 Sesto San Giovanni (MI), Italy; simone_donzelli@waters.com; Davide_Ambrogi@waters.com .

[5] Waters Corporation, Scientific Operations, Wilmslow SK9 4AX, United Kingdom; Heather_A_Brown-Manchester@waters.com; lisa_calton@waters.com

[6] Department of Psychological, Health and Territorial Sciences, "G. d'Annunzio" University of Chieti-Pescara, Chieti, Italy.

[§] These authors equally contributed to the work.

* Correspondence: claudia.rossi@unich.it ; Tel.: +39-0871-541596.

Abstract: The introduction of Tandem Mass Spectrometry (MS/MS) to clinical laboratories and the advent of expanded Newborn Screening (NBS) has been a crucial change to public health programs worldwide. Speed, robustness, accuracy, selectivity, and specificity of analysis are all requirements of expanded NBS and are needed to minimize false positive results risks, to possibly eliminate false negatives and to improve the positive predictive value of NBS. In this study, we firstly evaluated the analytical performances of the RenataDX Screening System, a fully integrated flow-injection MS/MS (FIA-MS/MS) IVD system for high-throughput dried blood spot (DBS) analysis in a routine NBS laboratory. Since a choice of several commercial NBS kits is available, we sought to compare NeoBase™ 2 (PerkinElmer®) and MassChrom® (Chromsystems) non-derivatized kits on the RenataDX platform by evaluating their analytical performances. Moreover, we verified the degree of correlation between data obtained by the two different NBS MS/MS kits by FIA-MS/MS of over 500 samples. Our data suggests that both the methods well correlate, having clinically insignificant differences that do not impact the NBS result. Finally, while NeoBase™ 2 offers an easier and faster sample preparation, MassChrom® provides a cleaner sample extract which empirically should improve instrument reliability.

Keywords: metabolomics; newborn screening; metabolites; high-throughput omics technologies; mass spectrometry; inborn errors of metabolism, analytical evaluation.

1. *Methods used in Analytical Performance Assessment between ACQUITY UPLC I-Class and RenataDX Systems*

The intra-day precision was evaluated by six replicate analyses of four QCs levels (A, B, C, D) provided by CDC and estimating the coefficient of variation (CV) in terms of percentage as the ratio of the standard deviation (SD) to the mean value multiplied by 100. The inter-day precision was quantified as %CV of each QC level provided by CDC measured in singleton in batches over a 5 day period. Repeatability test was also performed through the analysis of QC materials from the NeoBase™ 2 Non-derivatized MSMS kit, quantifying the %CV of 18 replicate analyses at two different concentration levels per analyte.

The correlation coefficient R^2 for each analyte, as an estimate of the degree of agreement of the two methods, was calculated as the square of the Pearson correlation coefficient derived from the equation linking the data obtained by the ACQUITY UPLC I-Class-MS analysis as X variables to the ones quantified by the RenataDX-MS analysis as Y variables. In addition, assuming that each plate containing DBS samples for FIA-MS/MS analysis had its first wells consequently occupied by blank, low QC, high QC and blank (Figure S3), carryover was measured as the ratio of the difference between the blanks to the high QC multiplied by 100. The carryover was calculated for at least 10 plates analysed by the two systems in comparison.

Table S1. Comparison of intra-day precision values from CDC QC levels between ACQUITY UPLC I-Class and RenataDX Screening systems. For each analyte, the lower %CV value from the comparison for each QC level analysed by the two analytical systems was underlined.

Intra-day	ACQUITY				RenataDX			
	**Level A**	**Level B**	**Level C**	**Level D**	**Level A**	**Level B**	**Level C**	**Level D**
ARG	2.74	5.18	6.44	4.39	5.70	3.98	4.34	6.50
CIT	8.95	5.22	7.12	9.25	9.69	3.65	8.55	7.46
LEU\ILE\PRO-OH	3.85	4.92	5.51	4.08	5.30	3.57	4.90	5.26
MET	6,04	4.62	5.76	5.68	7.41	7.66	4.91	6.13
PHE	3.99	4.96	4.74	4.17	5.09	4.23	6.33	5.70
TYR	6.31	6.27	5.40	5.03	8.98	4.35	3.84	5.73
VAL	5.01	4.60	4.89	5.26	4.78	4.61	4.82	4.04
C0	4.67	2.10	4.93	6.00	6.57	4.98	4.40	4.39
C10	7.90	6.78	6.83	5.78	4.23	4.50	5.81	5.58
C2	5.21	4.39	6.49	4.48	5.72	3.00	5.82	5.18
C3	7.55	7.16	5.43	5.12	8.12	5.56	5.52	4878
C4	7.75	2.97	4.89	6.56	5.42	5.45	6.05	5.05
C5	7.45	6.57	6.51	7.98	6.07	5.52	6.12	4.93
C5DC\C6OH	21.92	10.07	6.46	6.76	7.78	7.66	3.10	7.26
C6	15.71	2.64	7.47	4.46	9.41	5.98	7.94	3.94
C8	6.99	5.53	4.46	6.68	9.36	6.32	5.83	6.34
C12	6.73	4.52	6.93	6.33	8.28	5.25	5.68	6.42
C14	11.06	4.62	4.83	6.31	6.16	4.77	5.24	5.70
C16	5.63	5.38	5.49	5.14	7.15	5.44	6.55	4.60
C18	6.57	5.49	5.25	5.21	5.08	5.62	4.64	6.26

Table S2. Comparison of inter-day precision values from CDC QC levels between ACQUITY UPLC I-Class and RenataDX Screening systems. For each analyte, the lower %CV value from the comparison for each QC level analysed by the two analytical systems was underlined.

Inter-day	ACQUITY				RenataDX			
	**Level A**	**Level B**	**Level C**	**Level D**	**Level A**	**Level B**	**Level C**	**Level D**
ARG	7.07	6.81	8.60	5.69	6.89	6.45	7.19	4.48
CIT	12.8	9.74	8.51	6.85	8.67	8.41	10.89	4.49
LEU\ILE\PRO-OH	3.24	6.11	9.04	4.30	4.74	5.21	8.18	4.56
MET	6.19	4.40	7.39	4.98	5.83	3.14	4.89	4.27
PHE	4.19	6.34	6.26	3.61	3.12	5.71	8.10	5.26
TYR	6.33	6.18	8.00	5.14	8.55	4.23	6.19	4.38
VAL	4.86	5.99	7.78	3.36	3.77	4.98	7.10	4.35
C0	5.03	3.32	7.73	2.48	6.43	5.54	5.39	5.28
C10	8.43	4.83	9.17	4.04	6.52	7.01	9.41	4.48
C2	4.76	6.97	8.97	3.93	4.28	5.55	8.90	3.35
C3	3.56	4.51	8.46	6.10	7.44	3.65	7.26	6.21
C4	7.40	5.18	8.75	6.13	4.90	6.14	7.76	5.71
C5	13.49	10.08	9.69	6.37	5.84	5.71	9.41	5.92
C5DC\C6OH	19.54	5.45	7.93	8.85	9.64	2.61	6.05	6.25
C6	5.82	7.16	11.74	3.55	6.32	6.63	9.85	3.90
C8	7.09	6.67	8.47	3.71	6.36	10.00	7.83	3.45
C12	5.73	5.82	8.47	6.03	3.35	7.73	8.39	4.40
C14	0	5.93	8.51	5.49	8.86	6.75	6.76	5.57
C16	4.53	6.10	8.87	3.69	6.38	4.55	7.80	4.78
C18	3.94	4.76	7.28	5.08	4.76	6.13	7.05	4.37

Table S3. Comparison of carryovereffects between ACQUITY UPLC I-Class and RenataDX Screening systems. For each analyte, the lower carryover valuewas highlighted in bold.

	**ACQUITY**	**RenataDX**
ARG	0.254	**0.003**
CIT	0.003	**-0.023**
LEU\ILE\PRO-OH	0.043	**0.023**
MET	0.128	**3.2E-05**
PHE	0.141	**0.024**
TYR	0.220	**0.020**
VAL	0.099	**0.056**
C0	0.019	**0.004**
C10	**0**	0.004
C2	0.021	**0.005**
C3	0.015	**0.002**
C4	-0.042	**0.004**
C5	**0**	-5.2E-05
C5DC\C6OH	**0**	0.001
C6	-0.206	**0.015**
C8	**0**	0.007
C12	**0**	-0.001
C14	**0**	-0.005
C16	0.055	**0.078**
C18	**0**	0.484

Table S4. R2 values from correlation analysis of DBS samples between ACQUITY UPLC I-Class and RenataDX Screening systems.

	**R2**
ARG	0.982
CIT	0.892
LEU\ILE\PRO-OH	0.995
MET	0.945
PHE	0.995
TYR	0.977
VAL	0.990
C0	0.987
C10	0.966
C2	0.994
C3	0.992
C4	0.989
C5	0.953
C5DC\C6OH	0.778
C6	0.862
C8	0.904
C12	0.986
C14	0.987
C16	0.988
C18	0.994

Table S5. Comparison of carryover effectsby NeoBase™ 2 and MassChrom® non-derivatized kits on theRenataDX Screening System. For each analyte, the lower carryover value was shownin bold.

	**NeoBase™ 2**	**MassChrom®**
ARG	0.3958	**-0.035**
CIT	0.214	**-0.022**
LEU\ILE\PRO-OH	0.125	**0.011**
MET	0.137	**0.052**
PHE	0.124	**-0.002**
TYR	0.162	**0.131**
VAL	0.217	**0**
C0	0.096	**-0.003**
C2	0.095	**0.001**
C3	0.080	**-0.001**
C4	0.092	**0.005257**
C5	0.089	**0.029**
C5DC\C6OH	0.086	**0.024**
C6	0.083	**0.018**
C8	0.091	**0**
C10	0.044	**-0.013**
C12	0.047	**0.041**
C14	0.055	**0.038**
C16	0.083	**0.015**
C18	0.080	**0.007**

Table S6. MS parameters used for FIA-MS/MS analysis by NeoBase™ 2 Non-derivatized MSMS kit. For each analyte, MRM transition, cone voltage (V) and collision energy (eV) are shown.The internal standards (ISs) are reported in bold.

**Abbreviations of analytes and ISs**	**Analyte**	**Transition**	**Cone potential**	**Collision energy**
Arg<br>**$^{2}H_{4}$, ^{13}C-Arg**	Arginine	175.1>70.1<br>180.1>75.1	34	21
Cit<br>**2H_{2}-Cit**	Citrulline	176.1>113.1<br>178.1>115.1	24	16
Leu/Ile/Pro-OH<br>**$^{2}H_{3}$-Leu**	Leucine/Isoleucine/Hydroxyproline	132.1>86.1<br>135.1>89.1	24	10
Met<br>**$^{2}H_{3}$-Met**	Methionine	150.1>104.1<br>153.1>107.1	24	10
Phe<br>**$^{13}C_{6}$-Phe**	Phenylalanine	166.1>120.1<br>172.1>126.1	25	12
Tyr<br>**$^{13}C_{6}$-Tyr**	Tyrosine	182.1>136.1<br>188.1>142.1	26	12
Val<br>**$^{15}N_{2}$-$^{13}C_{5}$-Val**	Valine	118.1>72.1<br>124.1>77.1	23	10
C0<br>**$^{2}H_{9}$-C0**	Free carnitine	162.1>1030<br>171.2>103.0	38	16
C2<br>**$^{2}H_{3}$-C0**	Acetylcarnitine	204.1>85.0<br>207.1>85.0	34	18
C3<br>**$^{2}H_{3}$-C3**	Propionylcarnitine	218.1>85.0<br>221.2>85.0	32	18
C4<br>**$^{2}H_{3}$-C4**	Butyrylcarnitine	232.2>85.0<br>235.2>85.0	36	18
C5<br>**$^{2}H_{9}$-C5**	Valerylcarnitine	246.2>85.0<br>255.2>85.0	38	20
C6<br>**$^{2}H_{3}$-C6**	Hexanoylcarnitine	260.2>85.0<br>263.2>85.0	37	20
C5DC/C6OH<br>**$^{2}H_{6}$-C5DC**	Glutaryl-carnitine/3-Hydroxy-hexanoylcarnitine	276.2>85.0<br>282.2>85.0	40	24
C8<br>**$^{2}H_{3}$-C8**	Octanoylcarnitine	288.2>85.0<br>291.2>85.0	42	22
C10<br>**$^{2}H_{3}$-C10**	Decanoylcarnitine	316.2>85.0<br>319.3>85.0	45	22
C12<br>**$^{2}H_{3}$-C12**	Dodecanoylcarnitine	344.3>85.0<br>347.3>85.0	46	24
C14<br>**$^{2}H_{3}$-C14**	Tetradecanoylcarnitine (myristoylcarnitine)	372.3>85.0<br>375.3>85.0	52	25
C16<br>**$^{2}H_{3}$-C16**	Hexadecanoylcarnitine (palmito-ylcarnitine)	400.3>85.0<br>403.4>85.0	55	26
C18<br>**$^{2}H_{3}$-C18**	Octadecanoylcarnitine (stearoylcarnitine)	428.4>85.0<br>431.4>85.2	56	28

Table S7. MS parameters used for FIA-MS/MS analysis by MassChrom® Amino Acids and Acylcarnitines from Dried Blood/Non derivatised LC-MS/MS kit. For each analyte, MRM transition, cone voltage (V) and collision energy (eV) are shown. The internal standards (ISs) are reported in bold.

Analyte	Transition	Cone	Collision Energy
Arg<br>**D7-Arg**	175.2>70.1<br>182.2>77.01	30	21
Cit<br>**D2-Cit**	176.1>113.1<br>178.1>115.1	22	15
Leu<br>**D3-Leu**	132.1>86<br>135.1>89	22	10
Met<br>**D3-Met**	150.1>133.1<br>153.1>136.1	22	10
Phe<br>**D5-Phe**	166.1>120.1<br>171.1>125.1	22	14
Tyr<br>**D4-Tyr**	182.1>136.1<br>186.2>140.1	24	14
Val<br>**D8-Val**	118.1>72.1<br>126.1>80.1	22	10
C0<br>**D9-C0**	162.2>85<br>171.1>85	35	20
C2<br>**D3-C2**	204.1>85<br>207.1>85	32	20
C3<br>**D3-C3**	218.1>85<br>221.1>85	32	18
C4<br>**D3-C4**	232.2>85<br>235.2>85	32	18
C5<br>**D9-C5**	246.2>85<br>255.2>85	40	23
C5DC<br>**D6-C5DC**	276.2>85<br>282.2>85	35	23
C6<br>**D3-C6**	260.2>85<br>263.2>85	35	22
C8<br>**D3-C8**	288.3>85<br>291.1>85	40	22
C10<br>**D3-C10**	316.3>85<br>319.3>85	40	24
C12<br>**D3-C12**	344.3>85<br>347.3>85	45	26
C14<br>**D3-C14**	372.3>85<br>375.3>85	50	27
C16<br>**D3-C16**	400.4>85<br>403.4>85	50	29
C18<br>**D3-C18**	428.4>85<br>431.4>85	55	29

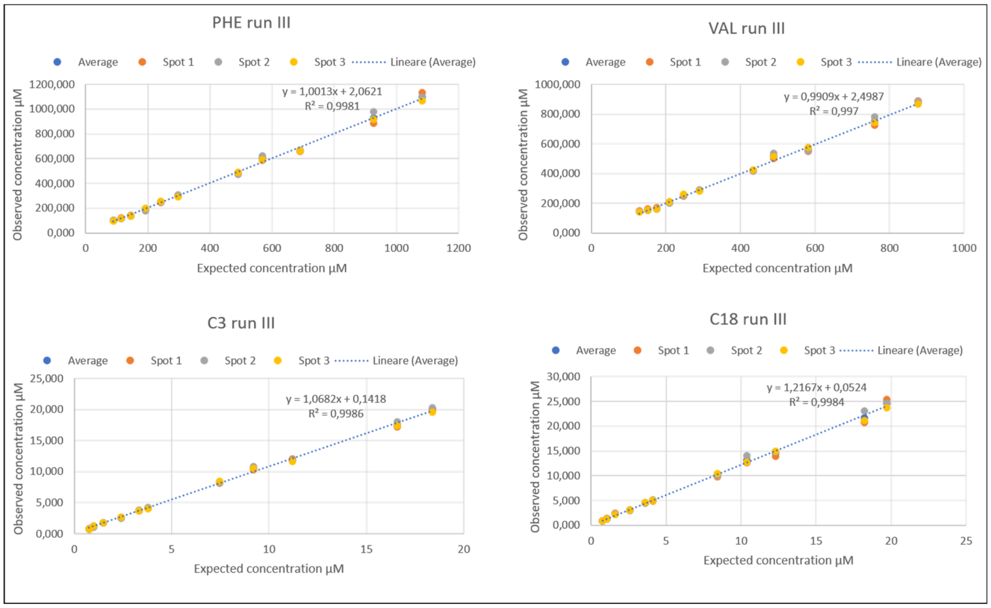

Figure S1. Linearity curves from the analysis of the third replicate of Phe and Val as aminoacids, C3 and C18 as acyl-carnitines, showing the equation of the linear regression curves and their respective R^2 values.

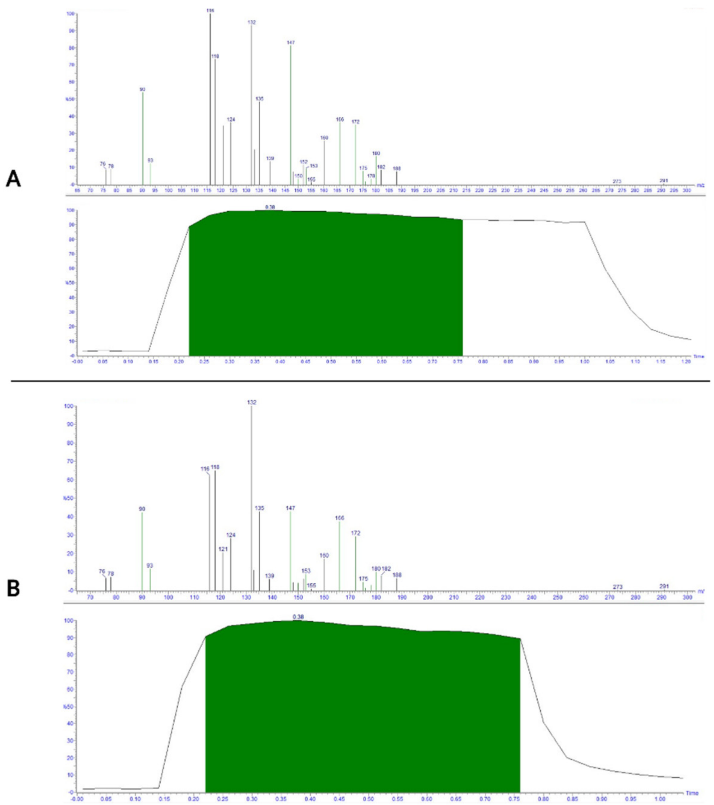

Figure S2. Examples of a typical flow injection chromatogram obtained by analyzing a low quality control (LC) provided by NeoBase™ 2 Non-derivatized MSMS kit on the RenataDX Screening System (A) and the ACQUITY UPLC I-Class System (B).

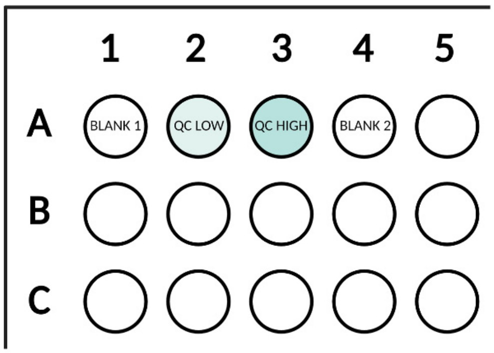

Figure S3. Zoom of a canonical 96-well plate to show how it is prepared for NBS, starting with a first blank that is immediately followed by low QC, high QC and a second blank. Carryover is estimated considering the blanks just mentioned and the high QC. Image created with BioRender.com.
